# Estrogens—Origin of Centrosome Defects in Human Cancer?

**DOI:** 10.3390/cells11030432

**Published:** 2022-01-27

**Authors:** Miriam Bühler, Ailine Stolz

**Affiliations:** Department of Experimental Toxicology and ZEBET, German Federal Institute for Risk Assessment (BfR), German Centre for the Protection of Laboratory Animals (Bf3R), Max-Dohrn-Str. 8-10, D-10589 Berlin, Germany; miriam.buehler@bfr.bund.de

**Keywords:** centrosome, centrioles, centriole defects, centrosome amplification, mitosis, whole chromosomal instability, natural and synthetic estrogens, endocrine disruptor, cancer

## Abstract

Estrogens are associated with a variety of diseases and play important roles in tumor development and progression. Centrosome defects are hallmarks of human cancers and contribute to ongoing chromosome missegragation and aneuploidy that manifest in genomic instability and tumor progression. Although several mechanisms underlie the etiology of centrosome aberrations in human cancer, upstream regulators are hardly known. Accumulating experimental and clinical evidence points to an important role of estrogens in deregulating centrosome homeostasis and promoting karyotype instability. Here, we will summarize existing literature of how natural and synthetic estrogens might contribute to structural and numerical centrosome defects, genomic instability and human carcinogenesis.

## 1. Introduction

Centrosomes are evolutionarily conserved organelles that serve as main microtubule-organizing centers (MTOCs) of animal cells with a key role in mitotic spindle organization, faithful chromosome segregation, cell polarity, adhesion, motility, migration, and cilia formation (reviewed in [1]). Defects in centrosome integrity including structural and numerical alterations, can disturb centrosome function causing genomic instability and tumor development [2]. Indeed, supernumerary centrosomes are clinically relevant in almost all types of cancers [3]. Although several signaling pathways underlie the etiology of centrosome defects [4,5,6], specific upstream regulators are hardly known. Estrogens of natural or synthetic origin (i.e., xenoestrogens) became a global research interest in this context, not least due to their ubiquitous occurrence in consumer products and the environment [7]. Throughout life, humans are exposed to a complex mixture of different estrogenic substances via various routes, including the skin, ingestion of food, dust, water, and through inhalation of gases. Estrogens may interfere with any aspects of the endocrine system, i.e., by altering the regulation of hormone synthesis, secretion, transport, or specific hormone receptor binding [8]. It could therefore be no coincidence that accumulating experimental evidence links estrogen exposure to a variety of different diseases including cancer [8,9,10,11]. One prominent example of a synthetic estrogen implicated in the development of several endocrine related cancers is Bisphenol A (BPA), the most common industrial chemical plasticizer produced worldwide and a major environmental contaminant [12]. A growing number of in vitro and in vivo studies reported that natural and environmental estrogens including BPA disturb centrosome duplication, bipolar mitotic spindle formation, spindle microtubule attachment to kinetochores, and karyotype stability [13,14,15,16,17,18,19,20,21,22]. How estrogens could induce centrosome defects, genomic instability and cancer particularly, in hormonally regulated tissues, will be discussed in the following sections.

## 2. The Centrosome: One becomes Two

Centrosomes are micron-scale cellular structures, which are composed of a pair of centrioles surrounded by a complex matrix of yet more than 100 different proteins. Centrosomes need to be duplicated exactly once per cell cycle to ensure faithful cell division and genome integrity. Thus, both centrosome biogenesis and duplication follow a strict regulatory machinery that is coordinated with each other [4,23,24].

### 2.1. Centrosome Architecture

The mammalian centrosome comprises two orthogonally arranged centrioles embedded in a proteinaceous matrix, termed the pericentriolar material (PCM). The PCM is a dense mass of a multitude of different proteins required for microtubule (MT) nucleation, organization and cellular signaling [25,26]. From hundreds of proteins that localize to the PCM, γ-tubulin ring complexes (γ-TuRCs) represent the main components of the PCM and act as microtubule nucleation templates. The highly structured PCM is organized into several layers, one localizes to the centriole wall and a second around. The inner layer functions to organize the PCM, whereas the outer layer provides docking sites for γ-TuRCs, thereby functioning in microtubule nucleation [27]. Important representatives for the inner layer of the PCM are Cep120, Cep192, and Cep152, as they function in centriole assembly [28]. A functional complex in the outer layer is formed by the scaffold proteins pericentrin/kendrin, CG-NAP/AKAP450, Cep192, CDK5RAP2, and Cep152, which target γ-TuRCs to the centrosome and function in PCM maturation, microtubule nucleation, anchoring, and positioning [26,29]. During interphase of the cell cycle, clustered, small electron-dense granules, i.e., centriolar satellites (CSs), surround the centrosome where they function as vehicles for protein trafficking towards the centrosome (reviewed in [30]). By the entry of mitosis, CSs gradually dissolve and become completely absent during metaphase of mitosis. Although the reason for mitotic dissolution is largely unknown, accumulating experimental evidence emerges that CSs may play an essential role for a variety of mitotic processes including mitotic spindle pole maintenance, centrosome duplication, microtubule organization and nucleation, among others [31,32]. Given PCM1 is one of the first CS proteins discovered, it has now become a widely accepted marker for centriolar satellites [32,33]. PCM1 granules are thought to act as scaffolding complexes to move centrosome-associated proteins, such as pericentrin, ninein, Cep90, and Cep290, along microtubules to the PCM, where they exert their specific functions [33,34,35,36]. Since PCM1′s discovery, many more proteins have been identified as CS components including Cep72, Cep90, Cep131, and the Coiled-Coil Domain Containing 13 (CCDC13). They are thought to regulate microtubule nucleation capacity and maintain centrosome integrity [35,37,38].

**Figure 1:**
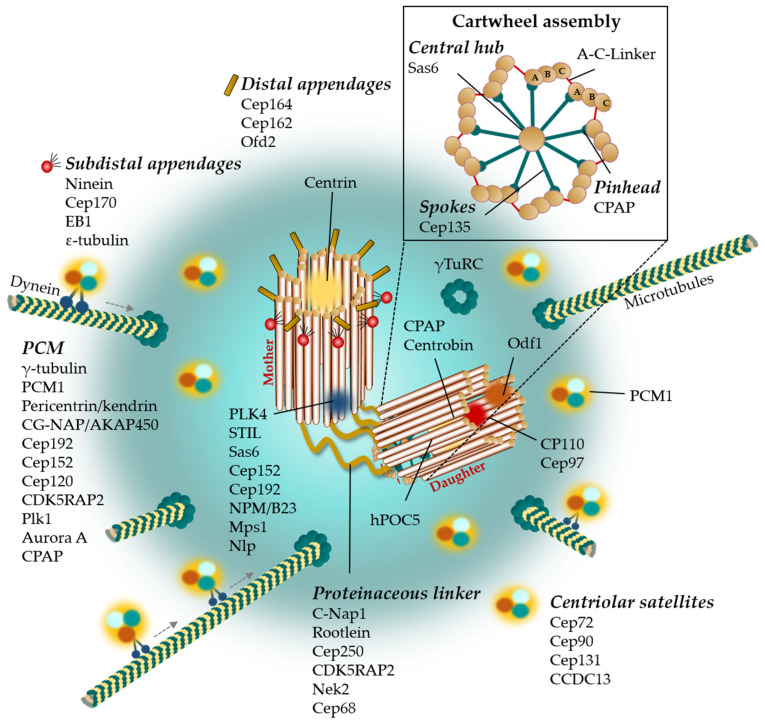
Mammalian Centrosome Architecture. The Centrosome consists of two orthogonal centrioles, embedded in the pericentriolar material (PCM) and is surrounded by small electron-dense granules (i.e., centriolar satellites). Centriolar satellites and PCM proteins are recruited towards the centrosome for centriole and microtubule assembly. Spindle microtubules are nucleated from γ-tubulin ring complexes (γ-TuRCs). The mother centriole is characterized by its sub-distal and distal appendages, whereas the daughter centriole contains a characteristic cartwheel structure. The centriolar wall consists of microtubule triplets of A-, B, and C-tubules.

Centrioles have a characteristic barrel shape of nine sets of triplet MTs. Each MT triplet is composed of A-, B-, and C-tubules that contain protofilaments of α- and β-tubulin heterodimers, whereby A- and C-tubules interconnect by a so-called ‘A-C-Linker‘ [39]. The calcium-binding EF-hand protein centrin, localizes at the distal lumen of the centrioles and represents a well-accepted centriole marker. Its function is not fully understood but centrin is thought to play a crucial role in centriole duplication and separation [40,41,42]. A striking feature of the mature (i.e., mother) centriole is the formation of sub-distal and distal appendages, which are essential for anchoring the centriole to the plasma membrane [43]. Important components of the sub-distal appendages are ε-tubulin, EB1, ninein and Cep170, among others. ε-tubulin is involved in centriole assembly [44,45,46], whereas ninein and EB1 are essential for MT minus-end anchoring at the centrosome [36,47]. Cep170 plays a role in MT organization and cell morphology [48]. At the distal appendages, Cep164 and Cep162 are required for PCM assembly and maintenance [49,50]. The younger, daughter centriole, harbors a characteristic cartwheel structure that dictates the nine-fold symmetry. The cartwheel defines the inner lumen of the centriole. It is composed of a central hub surrounded by nine spokes, each of which is emitted in the direction of the peripheral microtubule triplets [24,51,52]. The spindle assembly abnormal protein 6 (Sas-6) is a key component of the center of the cartwheel and interacts with Cep135, which functions as a physical linker between the central hub and the microtubule triplets by forming the spokes and pinheads [53]. The complex architecture of the mammalian centrosome along with its most important molecular structures and involved proteins are summarized in Figure 1.

### 2.2. Centrosome Duplication Cycle

To ensure faithful mitotic spindle assembly and chromosome segregation, the cell needs to control two events: First, centriole duplication only once per cell cycle and second, the assembly of only one new centriole per pre-existing one. The centrosome duplication cycle occurs in concert with the eukaryotic cell-division cycle, thereby sharing members of three main families of serine/threonine kinases, i.e., members of the cyclin-dependent protein kinases (CDKs), the polo-like kinases (Plks), and the Aurora kinases. In late G1, cyclin E activates CDK2, initiating DNA synthesis as well as centrosome duplication, thus coupling these two events [54,55]. The physical separation of the two centrioles (termed centriole disengagement) marks the start of centrosome duplication. Together with separase, Plk1 triggers disengagement of the mother and daughter centriole. Once parental centrioles are separated, a loose connection remains between their proximal ends and helps to focus the MT-organizing activity of the centrosomes. This type of connection, termed ‘G1-G2 tether’ (GGT), is composed of C-Nap1, Cep250 and rootletin [56] as well as Cep68 and CDK5RAP2 [57]. At the G1 to S transition of the cell cycle, one new centriole (also called procentriole) is formed orthogonally to each pre-existing parental (mother) centriole. The initiation of procentriole assembly requires the activity of CDK2/cyclin E and polo like kinase 4 (Plk4) [58,59]. CDK2 phosphorylates nucleophosmin (NPM/B23), enabling Mps1 to localize to the centrosome, which seems to be a critical step in procentriole formation, as the removal of NPM/B23 and/or Mps1 causes centriole re-duplication [60,61]. Plk4 is recruited to the site of procentriole assembly by the scaffolding proteins Cep192 and Cep152 [62,63]. There, Plk4 recruits SCL-interrupting locus protein (STIL) and Sas-6, building the basics for the characteristic cartwheel structure [64,65]. Because the recruitment of Plk4 represents a critical step in centriole duplication, its stability is regulated by SCFβTrCP/ubiquitin-dependent proteolysis [66].

Procentriole assembly starts with the recruitment of centrosomal protein 4.1-associated protein (CPAP) by STIL and Sas-6 [67,68]. Once formed, procentrioles elongate during the S and G2 phase of the cell cycle. The newly formed procentriole and adjacent mother centriole are tightly connected by a ‘S-M linker’ (SML) that forms during S-phase and persists until centriole disengagement in late M phase of the cell cycle [69]. CPAP triggers γ-tubulin dependent nucleation of the A-tubules and their attachment to the cartwheel structure to build the procentriole wall [67,70]. In addition, centrobin localizes to the outside of the triplet microtubule blades of the daughter centriole [71] and interacts with CPAP and α/β-tubulin heterodimers to promote centriole elongation and to protect CPAP from degradation [72]. The length of the procentriole depends on the proteome of centriole 5 (hPOC5) and centriolar coiled coil protein 110 (CP110). hPOC5 continuously accumulates in the distal lumen of the procentriole to promote elongation [73]. By contrast, CP110 together with Cep97, acts as a capping structure to limit extension by localizing to the distal end of the centrioles [74]. Ofd1 regulates centriole length by complexing with α- and γ-tubulin at the distal ends of centrioles [75]. Acquisition of PCM proteins, such as pericentrin, and γ-TuRC marks centriole maturation, thus increasing the nucleation ability of the centrosome. Plk1 plays a key role in the initiation of centrosome maturation by phosphorylating pericentrin. Phosphorylated pericentrin in turn, triggers the recruitment of other scaffolding proteins, such as Cep192 and CDK5RAP2, γ-tubulin, Aurora A and also Plk1 itself [76]. Thereby, CDK5RAP2 stimulates microtubule nucleation by its association with γ-TuRC [57]. In addition, CPAP localizes to the PCM where it interacts and forms a scaffolding complex with PCM proteins such as γ-tubulin, thus, regulating PCM recruitment [77]. The formation of distal and sub-distal appendages, determining the parental centriole, completes the maturation process. At the end of G2, Aurora A activates Plk1, which phosphorylates the Hippo pathway effector kinase Mst2, triggering the binding to the NIMA-related kinase Nek2. Nek2 phosphorylates the proteinaceous (i.e., GGT) linker proteins, C-Nap1 and rootletin, thus separating the mature centrosomes [78]. Once the linker is dissolved, the two centrosomes move to opposite directions. CDK1 and Plk1 phosphorylate the plus end-directed motor protein Eg5 to stimulate its binding to MTs and to trigger the separation of the centrosomes through antiparallel MT sliding [79]. At the G2/M transition, Plk1 controls the recruitment of PCM proteins to the centrosome to enhance MT nucleation capacity [80]. At the onset of mitosis, Plk1 displaces the ninein-like protein Nlp from the mother centriole, which is a prerequisite for MT assembly mediated by the γ-TuRC and pericentrin [81]. Microtubules grow with their plus-end extending towards the equatorial plane of the cell and their minus-end pointing towards the centrosome. The structure and function of the spindle depends on microtubule-dependent motor proteins (MAPs), which bind to the wall of the microtubule [82]. With the separation of the procentriole from the parental centriole and the loss of the cartwheel structure towards the end of mitosis, a new centrosome duplication cycle starts.

## 3. Centrosome Defects on a Structural and Numerical Level

**Figure 2:**
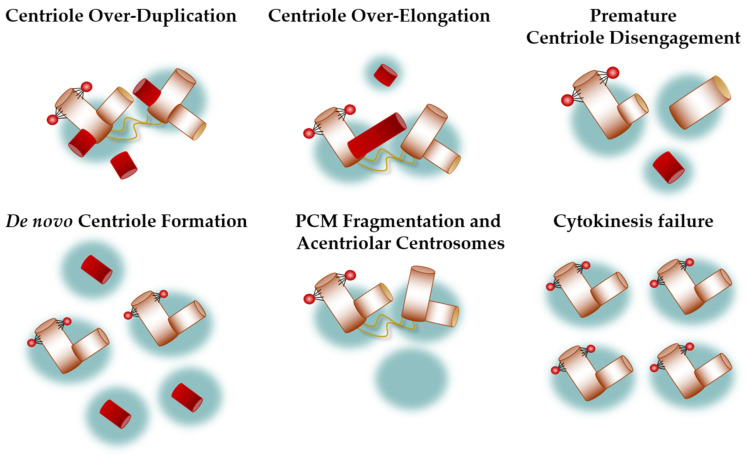
Origin of Centrosome Defects. Centrosome defects include structural and numerical changes. Structural defects include alterations in centriole size, length, or the amount of pericentriolar material, among others. Numerical changes are defined by more than two centrosomes per cell and are referred to as centrosome amplification (CA). Many mechanisms underlie the etiology of centrosomal alterations, these include centriole over-duplication, centriole over-elongation, premature centriole disengagement, de novo centriole formation, PCM fragmentation, and cytokinesis failure.

Defects in centrosome integrity and function have been associated with the pathogenesis of human cancers, particularly of hormonally regulated tissues such as breast, ovary, prostate, or testis, but also of lung, colorectal, and neural cancers, among others [3,6,83,84]. To understand the underlying mechanisms causing cancer to avoid potential risk factors for human health and to specify patient-specific therapies, cellular, molecular, and biomedical science focuses on the identification of possible upstream regulators disrupting centrosome homeostasis. Defects in centrosome integrity can be broadly classified into structural and numerical alterations, although structural alterations most likely result in numerical changes [84,85,86]. Structural defects include alterations in centriole size, length, or the amount of pericentriolar material, among others. Numerical changes are defined by more than two centrosomes per cell and are referred to as centrosome amplification (CA). A number of different mechanisms can trigger the loss of centrosome integrity and CA, mainly through dysfunction of centrosome-associated proteins, deregulation of the centrosome cycle, centriole-defects, or failure of cytokinesis [83,84]. A deeper understanding of the origin of centrosome abnormalities in cancer could have a powerful impact on the development of therapies and new biomarkers [86]. The following chapter will summarize major causes of centrosome alterations (Figure 2).

### 3.1. Centriole Over-Duplication

Centriole over-duplication leads to the formation of a rosette-like structure, composed of a mother centriole surrounded by multiple daughter centrioles. Initially, amplified centrioles remain connected to the mother and function as single entities within the first duplication cycle but consequently, form extra centrosomes in the following duplication rounds [58,87,88]. As mentioned before, Plk4 represents the key regulatory kinase of centriole duplication, being critical for the maintenance of correct centriole numbers. It is therefore not surprising that overexpression of *PLK4* causes centriole over-duplication, whereas depletion decreases centriole numbers. In detail, Plk4 induces the accumulation of multiple procentrioles on a single parental centriole [87,88]. Because excess centrioles assemble before mitosis, they remain engaged to the parental centriole and form a centriole rosette. However, descendant centrosomes with over-duplicated centrioles initiate centriole disengagement usually before the next duplication cycle, resulting in multiple centrosomes per cell [88]. Importantly, in addition to CA, centriole rosettes lead to an increase in MT nucleation and thus impair MT dynamics and further promote chromosomal instability (see below) [87]. Although Plk4-induced CA is thought to directly affect genomic stability and cause tumorigenesis [89], the interaction of CDK2 and the structural proteins CP110 and Sas-6 are required for this process, probably because they act as limiting modules for centriole assembly [58]. Another centrosome-associated kinase with a central function in centriole duplication is Aurora A. Overexpression of *AURKA*, which is frequently seen in tumors, is accompanied by CA, chromosome missegregation and aneuploidy, resulting in neoplastic transformation [20,90]. Although it is well-known that Aurora A functions in centrosome maturation and separation, as well as nucleation of MTs [91], the mechanism underlying Aurora A-mediated CA is still not fully elucidated. A study by Lukasiewicz et al. demonstrated that the phosphorylation of centrin seems to be crucial for Aurora A-induced CA [92]. Centrin is located at the centrioles where it controls centriole duplication [41]. Thus, overexpression of phosphorylated centrin results in increased centriole-like structures. The authors hypothesized that an increased stability of centrin would affect centriole formation or may cause a configuration that affects its interaction with other centriolar proteins, which finally causes centriole over-duplication [92].

In addition to major centrosome-associated kinases such as Plk4 and Aurora A, tumor suppressor proteins normally involved in the DNA damage response pathway, such as p53 and BRCA1, have also been shown to be involved in centriole over-duplication. p53 functions in G1/S and G2/M checkpoint-control and transcriptionally regulates several downstream targets, including *PLK4* expression [59,93,94]. Loss or mutational inactivation of *TP53* is highly associated with increased centrosome numbers [95,96]. Mechanistically, p53 activates p21 expression mainly during G1-S transition, when centriole duplication begins. In cells lacking p53, CDK2/cyclin E is free of p21-mediated inhibition, allowing excess centriole re-duplication in one cell cycle. Similarly, inhibition of p21 has been shown to cause the accumulation of extra centrosomes with multiple centrioles [97]. There seems to be a strong association between *TP53* mutation, *CCNE1* overexpression and the induction of CA. In fact, it appears that p53 and cyclin E synergistically induce CA [96]. The exact role of how cyclin E-CDK2 regulates centriole numbers is still unclear, yet there are several hypotheses. Although cyclin E activates CDK2 at the G1/S transition, overexpression of *CCNE1* results in accumulation of S and G2/M cell populations and a delay in mitosis due to unaligned chromosomes in prometa- and metaphase [98]. NPM, Mps1, CP110 and STIL represent prominent substrates of CDK2 [60,61,99]. CP110 is known to function as a capping device to control centriole length (see Section 2.2) and may positively regulate centrosome duplication or suppress premature centrosome separation [100]. In addition, overexpression of *CDK2* inhibits the STIL degradation pathway, thus leading to accumulation of STIL at the centrosome and subsequent initiation of centriole over-duplication [99]. Another important mediator of centriole over-duplication is the tumor suppressor protein BRCA1, normally functioning in homology-directed DNA repair and transcriptional regulation (reviewed in [101]). BRCA1 is thought to regulate centriole numbers by ubiquitination of centrosome-associated proteins, especially of γ-tubulin, and inhibition of BRCA1 function results in multiple centrioles [102]. CA originating from centriole over-duplication was also demonstrated for the high-risk human papillomavirus (HPV) types 16 and 18 carrying the two oncogenes E6 and E7. E6 subverts multiple mitotic checkpoints by inactivating p53, forcing cells to bypass a damage-induced G1/S arrest, resulting in centriole re-duplication. By contrast, E7 could induce CA by uncoupling the centrosome duplication cycle from the eukaryotic cell cycle, leading to increased procentriole formation [103] and disruption of normal centriole duplication by increasing *PLK4* mRNA steady-state levels [104].

It appears that centriole over-duplication is one of the principal mechanisms causing CA as shown by studies of solid tumors, hematologic malignancies, and human melanoma cell lines [87,105]. The majority of aberrant centrosomes in primary human malignancies showed centriole rosettes, which represent early stages of centriole over-duplication [87]. In human melanoma cells, only a small number of centrosomes harbored mother centrioles, indicating that CA predominantly arises through centriole over-duplication [105].

### 3.2. Centriole Over-Elongation

Overly long centrioles induce CA through mechanisms involving centriole fragmentation and ectopic procentriole formation. Newly formed procentrioles elongate throughout the S and G2 phase of the cell cycle. Normally, centriole length is tightly controlled, but changes in gene expression of centrosomal proteins or altered transcriptional modifications that control centriole structure, can lead to over-elongated centrioles [74,106]. Thus, overexpression of *CPAP* and decreased *CCP110* cause overly long centrioles [74,106]. In line with this, the absence of CPAP prevents centriole duplication, thereby causing asymmetrical distribution of the PCM between both centrosomes during mitosis [106]. Overexpression of *CPAP* however, results in excess procentrioles and disorganized centrosomes. Mechanistically, abnormally elongated centrioles recruit PCM components, facilitate the formation of supernumerary procentrioles, and ultimately induce serious defects in centrosome architecture. Amplified centrosomes cause the formation of transient multipolar mitotic spindles, impairing the fidelity of cell division with disastrous consequences for genomic stability [106]. In fact, a systematic survey of centrosome abnormalities in the NCI-60 panel of human cancer cell lines found the deregulation of centriole length to be a common lesion in cancer. The authors observed that centriole over-elongation results in the generation of enlarged centrosomes that harbor increased MT nucleation capacities [107]. Another study comparing the proximal and distal parts of the centrioles in human cells using expansion microscopy showed that the two parts were of different lengths. Because the authors used HeLa cells, in which centriole duplication defects are rare, they concluded that the variabilities in centriole length are within the physiological limits of human cells and that there appears to be no monitoring mechanism to tightly control centriole length. Consequently, in the absence of a control mechanism, centrioles are probably prone to over-elongate beyond their physiological tolerable range. Indeed, analysis of centriole elongation dynamics in different cell cycle phases revealed that procentrioles drastically over-elongate at mitotic arrest. These results demonstrate that over-elongated centrioles can arise even without chemical or genetic manipulation of centrosome-associated proteins and probably by any event that causes mitotic delay [108].

### 3.3. Premature Centriole Disengagement

Progression through the centrosome cycle requires the generation and dissolution of two substantially different types of connections between centrioles (see Section 1.2, [69]). The physical separation of the mother and daughter centriole represents an important licensing step in centriole duplication ensuring that centrosomes are duplicated only once per cycle. However, premature centriole disengagement can lead to numerical and structural centrosome abnormalities. Indeed, early disconnection of centrioles in interphase may trigger re-duplication of centrioles, resulting in an excess of centrosomes [109]. By contrast, the loss of centriole cohesion before completion of chromosome segregation results in excess centrosomes with only one centriole [110]. Only little is known about loss of centriole cohesion, but several centrosome-associated proteins have been identified to be potentially involved. Given C-Nap1 is an indispensable linker component, its absence induces centriole disengagement independent of the cell cycle phase. This suggests that interference with C-Nap1 function promotes the separation of parental centrioles, whether or not they are associated with growing procentrioles, allowing re-duplication of procentrioles more than once per cycle [111,112]. The same is observed in cells that overexpress *NEK2*. Centriole separation is dependent on Nek2-dependent phosphorylation of C-Nap1. Over-active kinase activity of Nek2 results in split centrioles in interphase, thus providing a condition of centriole re-duplication [113]. Moreover, partial repression of *PCNT* (encoding pericentrin) causes disengagement of centrosomes in interphase by negatively regulating Nek2 [114]. Astrin (Sperm-Associated Antigen 5, SPAG5) is thought to contribute to the regulation of separase activity because *SPAG5*-depleted cells showed multipolar spindles containing only one centriole, indicating that centrioles were separated too early in the cell cycle [115]. Recently, Wilhelm et al. reported premature centriole disengagement as an important origin of CA, at least in colorectal and breast cancer cells. Here, the majority of spindle poles of multipolar cells harbored only one centriole. The authors demonstrated that the disengagement process depends on ATR, Cdk1 and Plk1 activity in G2-phase [116].

### 3.4. De Novo Centriole Formation

New procentrioles normally require a template to assemble, i.e., the characteristic cartwheel structure that is exclusively present at the proximal end of mature centrioles [51]. For a long time, it was assumed that de novo assembly of centrioles occurs exclusively in a number of specialized cell types. However, studies on CHO and HeLa cells uncovered the contrary [117,118]. Khodjakov and colleagues demonstrated that removal of centrosomes in S-phase arrested CHO cells leads to de novo formation of centrioles. In contrast to procentriole assembly at the cartwheel template, where only one centriole is formed per parent centriole, the number of centrioles generated de novo seems to be arbitrary [118]. A study on HeLa cells revealed a mechanism, where centrosome misdistribution during mitosis could trigger de novo centriole assembly in transformed cells, resulting in CA and driving tumorigenesis. Only transformed HeLa cells with functionally suppressed p53 and Retinoblastom-Protein (Rb) were able to pass through G1-phase in the absence of centrioles. Moreover, de novo centriole assembly occurs exclusively when all resident centrioles were absent, suggesting that centrioles might bear an activity that suppresses a de novo assembly pathway [117]. Another study on HeLa cells hypothesized that centriole duplication and the de novo assembly pathway originate from the same mechanism. The authors showed that the formation of daughter centrioles did not depend on the presence of mother centrioles but was initiated within the PCM. These findings might explain why the de novo pathway is inhibited in the presence of centrioles. The authors suggested that the PCM provides an environment that supports centriole assembly. Indeed, the overexpression of *PCNT*, which is not directly involved in centriole assembly, exaggerated the PCM and caused the formation of numerous daughter centrioles as well [119].

### 3.5. PCM Fragmentation and Acentriolar Centrosomes

The PCM surrounds the two centrioles and functions as a platform for protein complexes involved in organelle trafficking and mitotic spindle formation. Perhaps the most prominent role of the PCM is to nucleate MTs, which are required for spindle assembly, spindle positioning, and intracellular transport. Additionally, cytoplasmatic centriolar satellites concentrate at the PCM, where they function as protein trafficker and assist to maintain mitotic spindle poles [26,31]. Inappropriate function of these associated proteins has been shown to induce PCM fragmentation, thereby resulting in excess acentriolar centrosomes (centrosome devoid of centrioles). One protein that is involved in the fragmentation of PCM is ch-TOG (MT-stabilizing protein colonic hepatic tumor-overexpressed gene), a microtubule-associated protein that is a homolog of XMAP215 and co-localizes with γ-tubulin at the centrosome. There, ch-TOG potentially anchors MTs after their release from nucleation sites. Depletion of *CKAP5* (encoding ch-TOG) resulted in a greater number of γ-tubulin foci with a reduced average area, probably due to the loss of the MT anchoring function. Nucleation sites might detach from the PCM resulting in the generation of fragmented γ-tubulin and pericentrin foci as observed in *CKAP5*-depleted cells [120]. Moreover, centrosome integrity requires the functional interaction of Aurora A, TPX2 and Plk1. Thereby, TPX2 controls both the Plk1-dependent recruitment of γ-tubulin to centrosomes as well as the Aurora A-controlled centriole cohesion and integrity. Thus, depletion of either Aurora A or Plk1 kinase causes supernumerary spindle poles that lack centrioles [121]. The formation of acentriolar centrosomes in Aurora A-depleted cells was further shown to require ch-TOG and MCAK (mitotic centromere-associated kinesin). Here, ectopic PCM fragmentation occurred after nuclear envelope breakdown in prometaphase. Aurora A-induced fragmentation might originate from unbalanced forces of kinetochores on spindle MTs, which generates excessive pressure on centrosomes resulting in loss of integrity [122]. Plk1 is further required for the association between the centrosomal protein Kizuna (Kiz) and the PCM component pericentrin. Depletion of *KIZ* caused the detachment of PCM components from the centrioles resulting in excess PCM fragments and spindle disorganization [123]. Another Kiz-interacting protein is Cep72, which targets Kiz, and PCM proteins such as γTuRC and CG-NAP/AKAP450 to the centrosome. The absence of Cep72 causes excess PCM foci, possibly through mislocalization of Kiz [124]. The presence of the centriolar satellite protein Cep90 has been shown to be essential for proper mitotic spindle integrity. Indeed, Cep90-depleted cells harbor excessive spindle poles devoid of centrioles [35].

Although only a few pathways for acentriolar centrosome formation have been identified so far, it seems to be an important marker for tumors, at least in breast cancer. In fact, a study on a cohort of 362 breast cancer patients revealed that acentriolar centrosomes might correlate or function as a marker for more aggressive tumors, as this phenotype was more prevalent in advanced stages [125].

### 3.6. Cytokinesis Failure

Cytokinesis defines the final part of cell division, in which the cytoplasm of a single cell divides into two daughter cells. Failures in cytokinesis occur when the formation or resolution of the cleavage furrow is disturbed, resulting in binucleate tetraploid cells with excess centrosomes. Members of the Aurora- and Polo-like kinase families are important regulators of cytokinesis. Overexpression of *AURKA*, *AURKB*, and *PLK1* was shown to cause the formation of binucleated cells that harbor supernumerary centrosomes [90], probably by a mechanism involving RhoA activation at the spindle midzone [126,127,128,129]. Other studies have revealed that CA occurs in cells with telomere-driven tetraploidy followed by mitotic slippage, endoreduplication or cytokinesis failure [130]. In *TP53* deficient cells, the induction of cells with supernumerary centrosomes and polyploidy increased to an even greater extent. These findings demonstrate that CA arises through a combination of cytokinesis failure and the incompetence to prevent polyploid cells from progressing through the cell cycle [90]. However, there is a growing body of experimental data showing that failure of cytokinesis does not result in stable centrosome amplification [131,132], suggesting that knowledge of tetraploidization as the origin of additional centrosomes may be incomplete. Thus, newly formed tetraploid cells artificially induced by cytokinesis failure rapidly lose extra centrosomes to maintain a near-tetraploid chromosome number because cells that inherit supernumerary centrosomes are more likely to undergo multipolar cell division. Consequently, tetraploid cells that inherit a single centrosome have a higher probability of long-term survival [131]. Although it is a popular model that tetraploidization promotes tumorigenesis through the accumulation of additional centrosomes, in light of current observations, it is possible that tetraploidization promotes tumorigenesis through means other than excess centrosomes, for example by increasing tolerance to genomic alterations or oxidative stress, genomic insults, and others [133].

## 4. Consequences of Centrosome Defects

Over a century ago, Theodor Boveri proposed a model where centrosome amplification causes severe mitotic defects leading to improper chromosome segregation, thereby driving tumorigenesis [134]. Although the role of aberrant mitosis in carcinogenesis has long been controversial, decades of research have now recognized abnormalities of the centrosome as a general feature in all major classes of human cancers [3,135].

### 4.1. Mitotic Laggards, Whole Chromosomal Instability, and Aneuploidy

Centrosomes organize the bipolar mitotic spindle to ensure high fidelity of chromosome segregation [1]. It is well accepted that structural and numerical centrosome alterations modify cell architecture due to malfunctions in MT nucleation and organization, which in turn, increase the frequency of chromosome missegregation [84,89,135]. Supernumerary centrosomes can prove lethal by causing the formation of multipolar mitotic spindles following anaphase catastrophe [136] or multipolar mitosis [137]. To overcome multipolar mitosis, cells have developed several coping strategies including inactivation of centrosomes, centrosome loss and centrosome clustering, to ensure bipolar mitotic spindle formation and cell survival [137]. While centrosome inactivation and loss seem to be prevalent in normal eukaryotic cells [138,139], it is unclear whether these mechanisms exist in tumor cells [140]. The best-studied and probably most common strategy to overcome cell death in the presence of multiple centrosomes is centrosome clustering (also termed coalescence), in which excess centrosomes are clustered into two groups to form a pseudo-bipolar spindle [137,140]. Centrosome clustering depends on a combination of cellular conditions that determine the fate of cells with extra centrosomes [137,141]. For example, functional components of the spindle assembly checkpoint (SAC), such as Mad2, Bub1 or Aurora B, are crucial to provide enough time for extra centrosomes to cluster, as they prevent the onset of anaphase at the presence of false attachments [137,142]. Since the interphase adhesion pattern and the distribution of actin-containing retraction fibers have a major impact on spindle orientation during mitosis [143], they appear even more essential for cells with supernumerary centrosomes. Indeed, cell-matrix adhesion- and actin-regulators are thought to organize excess centrosomes by affecting cortical force generators and their ability to pull astral MTs to induce centrosome movement [137]. In addition, two minus end-directed motor proteins, dynein [144] and HSET [145], have been shown to be significantly involved in centrosome clustering. Dynein is associated with transporting the spindle pole integrity protein, NuMa, to the minus ends of spindle MTs [146], whereas HSET functions in spindle elongation by crosslinking and sliding MTs [147]. By stabilizing the connection of centrosomes to the spindle pole, these motor proteins may increase the efficiency for centrosome clustering [144,145]. However, centrosome clustering is less effective, since it forces transient spindle multipolarity, with microtubules emanating from opposing spindle poles being attached to a single kinetochore [88]. This kind of attachment, termed merotely, is common in early mitosis and converted under normal conditions [148]. However, in the presence of supernumerary centrosomes, the cellular correction machinery appears to be overwhelmed. This leads to a dramatic increase in the frequency of merotelic attachments, forcing cells to enter anaphase before corrections are complete [88]. It is noteworthy that merotelic kinetochore attachments cause the generation of isolated, so-called lagging chromosomes during anaphase of mitosis. Since mitotic laggards are randomly distributed to the newly formed daughter cells, they represent an important precursor of whole chromosomal instability (w-CIN) (reviewed in [141,148]). W-CIN is defined as the process of ongoing gain or loss of whole chromosomes during mitosis, with supernumerary centrosomes and abnormal centrosome positioning as major underlying mechanisms [141,149]. Of note, w-CIN represents an important route to genome instability [150,151,152] and has been associated with cancer development and progression, as well as therapy resistance [153], ultimately leading to aneuploidy. In contrast to w-CIN, aneuploidy is the condition of abnormal chromosome numbers in a particular karyotype and accounts for 70–90% of cancer cells [154].

### 4.2. Impact on Cancer Development and Progression

Accumulating clinical evidence shows that defects of the centrosome are present in various precursor neoplasms and that more extensive alterations occur during the course of the disease [3]. The efficiency of centrosome clustering and by this, the frequency of w-CIN and aneuploidy, defines whether tumor progression is initiated or inhibited because low levels of w-CIN are thought to promote tumor development, whereas high levels of w-CIN might have protective roles [155]. In addition to w-CIN, supernumerary centrosomes followed by lagging chromosome formation can also promote DNA damage and chromosome rearrangements [150,152]. Consequently, CA leads not only to numerical chromosomal alterations but also to structural changes, compromising genomic integrity. Despite the fact that CA might contribute to tumorigenesis through w-CIN and aneuploidy, it is also recognized that additional centrosomes can affect cell physiology in ways unrelated to chromosome segregation. Indeed, supernumerary centrosomes are not only clustered in mitotic cells but also during interphase. How this occurs is unclear, but clustered centrosomes are able to recruit extra PCM, leading to enlarged centrosomes with an increased MT nucleation capacity. This, in turn, correlates with changes in cell shape and motility and could influence tumor architecture and the propensity of tumors to metastasize [156]. Increased MT nucleation capacity can greatly affect cellular physiology. For example, MTs regulate the disassembly of focal adhesions (FA) involved in cell migration [157], affect the activity of Rho GTPases that regulate cell invasion [158], and increase Rac1 activity and cell invasion [159]. It is therefore not surprising that an excess of centrosomes has been described in almost all types of cancers, such as breast, prostate, lung, ovarian or colon cancer ([3,6,160]; see Section 5.3). In fact, several studies report that CA is a causal and early event in breast tumorigenesis [83,135,161]. In a study of high grade breast adenocarcinomas, several structural alterations of centrosomes were observed, including an increase in number and size, as well as chaotic subcellular location and accumulations of PCM and centrioles [156]. Overexpression of *AURKA* and inhibition of the tumor suppressor BRCA1 are common lesions in breast cancer and associated with CA, w-CIN, and spontaneous tumorigenesis [162,163,164]. Similarly, the association between CA, w-CIN and *AURKA* overexpression has also been demonstrated in urothelial carcinoma. Here, CA and w-CIN cancers show a highly malignant behavior [96,165,166,167]. Support for CA in tumorigenesis was also demonstrated for prostate cancer, where the frequency of centrosome abnormalities correlating with w-CIN increased with progression of prostatic neoplasia to metastatic cancer [168,169]. The prognostic significance of CA in ovarian cancer is of clinical relevance as it is associated with higher stage, higher histologic grade, and more aggressive serous type compared with endometroid tumors [170]. Importantly, CA is not only relevant in endocrine-related tissues but also in other cancers such as gastrointestinal, neural, or bone cancer. Indeed, colorectal cancer represents a prime example for a tumor entity exhibiting w-CIN [171,172] with CA as a major underlying mechanism [173,174]. Thereby, CA is detected in low-grade dysplastic lesions and is more frequent in carcinoma [173,175]. Mutations of the tumor suppressor gene adenomatous polyposis coli (APC) are common in colorectal cancer and induce cytokinesis failure, tetraploidy and CA [176]. Aneuploid endothelial cells in solid tumors are highly associated with multiple centrosomes, and distinct γ-tubulin localization is also found in the cytoplasm of vascular endothelial cells, in areas of tumor angiogenesis in glioblastomas [177,178].

It is still a matter of controversy if CA is a cause or consequence of cancer progression. However, the observation of Pihan and colleagues that centrosomal abnormalities occur in pre-invasive carcinomas concomitantly with w-CIN, strengthen the idea that centrosome defects contribute to early stages of cancer and promote cancer progression [179]. Supporting this notion, the comparison of cytogenetic profiles of colorectal carcinoma cell lines revealed a correlation of chromosome segregation errors with abnormalities in centrosome structure, number, and function [180].

## 5. Estrogens—A Curse or a Blessing?

Environmental estrogens of natural or synthetic origin have become a global research interest not least because of their ubiquitous occurrence in consumer products (e.g., plastic bottles, cosmetics, food) and the environment (e.g., pesticides, biocides) [7]. Due to their interaction with estrogen receptors, estrogenic substances have different effects on human health, depending on the substance itself, its binding affinity, concentration, and exposure time. Substances with estrogenic activity are associated with a variety of diseases, including cancer [9,181]. Their occurrence and effects on human health are discussed in the following sections.

### 5.1. Natural Estrogens and Receptors

Estrogens control the reproductive cycle, and affect bone, skin, the cardiovascular system, and immunity. Given their physiological relevance, they are universally used as medications to regulate biological processes. Estrogens, including 17β-estradiol and its derivatives, estriol and estrone, together with progesterone, form the basis for combined oral contraceptives, hormone replacement therapies or for the treatment of some menstrual disorders [182]. 17β-estradiol, esterified estrogens or conjugated equine estrogens are used in combination with progestin primarily to treat menopausal and postmenopausal health risks [183]. Moreover, the estradiol metabolite 2-methoxyestradiol (2-MeOE2) is used as an antiangiogenic and anticancer agent [184]. Estrogens generally mediate their effects by binding to hormone receptors that trigger a cascade of biomedical responses, or directly to specific proteins involved in the control or release of hormones at specific target sites.

Estrogen signaling can be classified into genomic and non-genomic responses. Genomic responses are characterized by changes in gene transcription and are associated with ligand-activated transcription factors. By contrast, non-genomic responses include rapid signaling events, such as kinase activation or calcium mobilization. Genomic responses are considered to be the predominant mechanism of estrogen signaling and mainly involve the classical estrogen receptors (ER) -α and -β [185]. Binding of estrogens to ERs in the nucleus induces receptors to dimerize and bind to estrogen response elements (EREs) located in the promoters of target genes. Gene expression can also be indirectly regulated by ERs through protein–protein interactions with other classes of transcription factors [185]. Rapid non-genomic responses are mediated mainly by the alternative G-protein coupled receptor GPER1 [186]. When activated, heterotrimeric G proteins mobilize several effectors such as adenylyl cyclase (leading to cAMP production) or tyrosine kinase SCR. Activated SCR, in turn, leads to the activation of multiple effectors such as mitogen-activated protein kinase (MAPK), PI3K, or phospholipase C (PLC) and to the mobilization of calcium [187]. SCR further activates matrix metalloproteinases, leading to the release of free heparin-bound epidermal growth factor (HB-EGF) and consequent activation of epidermal growth factor receptor (EGFR) [188]. Peptide growth factors generally represent an important group of estrogen-independent ER activators [185]. Thus, estrogen and its receptors control a variety of physiological processes by altering gene expression or triggering fast biomedical cascades.

### 5.2. Environmental Estrogens and Estrogen Mimics

Environmental estrogens can be of natural or synthetic origin and are structurally and/or functionally related to endogenous estrogen. The most prominent classes of natural estrogens from the environment are phytoestrogens and mycoestrogens [189,190]. Flavonoids represent the most important subclass of phytoestrogens and are found in berries, cereals, nuts, soybean, and legumes, among others. Soy is the most important source of estrogenic flavonoids, with genistein and daidzein being the most prominent representatives [9]. Synthetic estrogenic compounds, on the other hand, are released into the environment through their use as herbicides (e.g., atrazine), pesticides (DDT and endosulfan), industrial chemicals (e.g., polychlorinated biphenyls (PCBs)), household products (e.g., nonylphenol and octylphenol), plastic products (e.g., bisphenol A (BPA) and phthalates), and pharmaceuticals (e.g., diethylstilbestrol (DES)). Because of their chemical properties, environmental estrogens are capable of accumulating in human tissues and therefore may affect the human body differently than endogenous estrogens [9,182,189]. Indeed, estrogenic substances can alter hormonal responses by interfering with other receptors, thereby inducing other cellular responses or altering the production of hormones [10,182,191]. When “an exogenous substance or mixture (…) alters function(s) of the endocrine system and consequently causes adverse health effects in an intact organism, or its progeny, or (sub) population”, it is defined by the World Health Organization (WHO) as an endocrine disrupting chemical (EDC) [8]. In order to protect human health and the environment from risks that may be posed by endocrine disrupting substances, the European Chemicals Agency (ECHA) collects and evaluates information on the properties and hazards of substances. Currently, 100 individual substances are listed on ECHA’s Endocrine Disruptor Assessment List (ECHA.eu, accessed on 5 January 2022 [192]), including Bisphenol A (BPA), one of the world’s most widely manufactured chemical plasticizers and a major environmental contaminant. To understand endocrine disruption, it is essential to understand their mode of action in the endocrine system and to apply appropriate methods to assess their impact [10,193].

### 5.3. Impact of Estrogens on Human Diseases and Cancer

The endocrine system is a communication network that controls important physiological processes such as growth, development, metabolism or reproduction [194]. It is therefore not surprising that many diseases can be attributed to imbalances in hormone secretion, regulation or metabolism. Prominent examples include disorders of growth and development, the thyroid, and bone metabolism, as well as cancers of the breast, ovary, prostate and testis [8,10,195]. Throughout life, humans are exposed to a complex mixture of natural and environmental estrogens, mainly through oral ingestion and inhalation of polluted air. Their effects on human health and their use are widely debated, as their inappropriate exposure can alter the hormonal and homeostatic system of the human body. On the one hand, phytoestrogens are generally considered to have beneficial effects, as they are used as preventive or therapeutic substances for various diseases such as osteoporosis, cardiovascular pathologies, or different types of cancer (reviewed in [9]). For example, the soy phytoestrogens daidzein and genistein have been shown to have neuroprotective properties [196,197,198]. Other benefits to the central nervous system include prevention and treatment of Alzheimer’s disease or depression [199,200]. Conflicting effects of phytoestrogens on breast cancer or menopausal symptoms are described, while there is strong evidence for a protective or preventive role in colorectal, endometrial, and prostate cancer, endometriosis, and osteoporosis (reviewed in [9]). Although the beneficial effects of phytoestrogens outweigh their risks, excessive consumption of phytoestrogens and their derivatives may be associated with adverse health effects [201].

Synthetic estrogens usually affect the reproductive system and neurodevelopment and can cause thyroid disorders and hormone-related cancers [8,10]. Probably the best known example is DES, a drug used in the 1940s to prevent miscarriage and premature births. Only later did it become apparent that the offspring of women treated with DES developed reproductive tract abnormalities and were at increased risk for cancer and other diseases [202,203,204]. In addition, one of the most commonly used synthetic estrogens, BPA, has been linked to a variety of human diseases and to alterations of hormone-sensitive organs, including the induction of hormone-related cancers of the breast, ovary, and prostate, even at low levels of exposure [12,205]. Importantly, animal studies suggest that fetuses and children are particularly vulnerable to BPA exposures [206]. Apparently, the timing of exposure is critical for the effect, as prenatal exposure to BPA and other endocrine active substances may impair social behavior such as communication, pair bonding, and social inquisitiveness or lead to altered susceptibility to tumor formation [207,208,209]. Several environmental estrogens have been associated with the induction of cell proliferation, transformation, and metastasis in endocrine-sensitive as well as non-sensitive tissues [210,211,212,213]. For example, BPA has been shown to promote migration, invasion, metastasis, proliferation, and cell transformation in lung, colon, and breast cancer cells, as well as in mammalian cells [211,212,213]. Similarly, DES has been shown to regulate cell proliferation in testis [214] and melanoma cells [215]. Another link between estrogens and tumor development arises from accumulating evidence reporting that estrogenic substances induce defects of the centrosome and w-CIN, which may contribute to tumor formation [17,20,216].

Overall, the role of environmental estrogens in the development of diseases and cancer is ultimately not entirely clear. Therefore, this exciting field of research certainly deserves attention in the future.

## 6. Estrogenic Loss of Centrosome Integrity and Genome Stability

**Figure 3:**
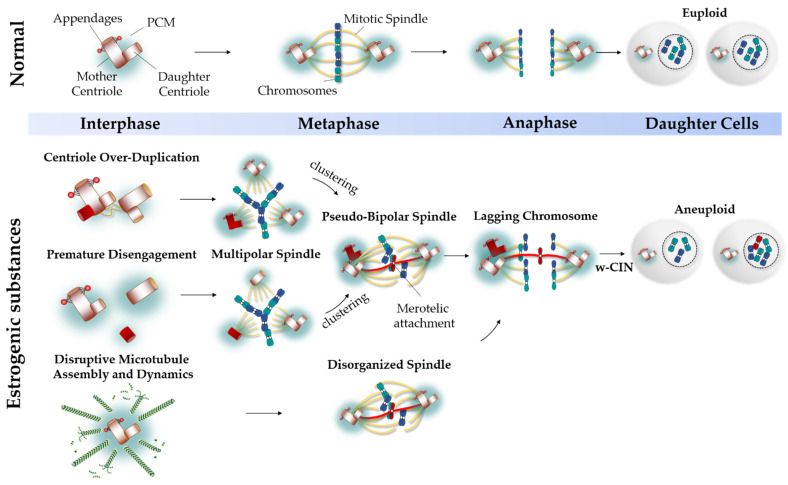
Estrogenic Loss of Centrosome Integrity and Genome Stability. In normal cells, centrosomes duplicate once and only once during the cell cycle, thereby ensuring equal chromosome segregation and formation of genetic identical euploid daughter cells. Structural or numerical centrosome aberrations are associated with whole chromosomal instability (w-CIN) and cancer progression. Environmental estrogens are able to alter steps of the centrosome cycle, thereby triggering defects in centrosome integrity. In the presence of estrogenic substances, centriole over-duplicate or disengage prematurely before completing chromosome segregation, forcing the generation of transient multipolar mitotic spindles with merotelic microtubule-kinetochore attachments. To avoid multipolarity-driven lethality, excess centrosomes are clustered into a pseudo-bipolar spindle, however, at the expanse of mitotic fidelity. Through binding to microtubules, estrogenic hormones disturb microtubule assembly and spindle dynamics, thereby causing defects in spindle organization that favor faulty kinetochore attachments. Finally, lagging chromosomes evolve at the equatorial plane of metaphase cells that manifest in w-CIN and aneuploidy, which represent hallmarks of human cancer.

Although several pathways underlie the etiology of centrosome defects in human cancers [3,84,86], the upstream regulators are hardly known. The first insights linking estrogens and estrogenic substances to centrosome defects and genome instability in cancers of hormonally regulated tissues came from studies in rodents, prostate, breast, and cervical (cancer) cells, in which sex hormones and environmental estrogens were reported to cause defects in bipolar mitotic spindle formation, centrosome duplication, spindle microtubule attachment to kinetochores, and increased karyotype variabilities [13,14,15,16,20,213]. How estrogens might be related to CA, w-CIN, and carcinogenesis, is discussed in the following sections (Figure 3).

### 6.1. Centriole Over-Duplication

In breast cancer cells, treatment with BPA resulted in disruption of the normal number of centrioles and multipolar mitotic spindles. Kim et al. [13] found that BPA impaired the expression and localization of mitotic regulators such as Plk1, HUPP, Kif2a, and TPX2, and centrosome-associated proteins including Plk4, STIL, and Sas-6. The authors demonstrated that BPA caused prolonged duration in prometa- and metaphase of the cell cycle and prolonged expression of several mitotic regulators such as Plk1, cyclin B1, and phosphorylated Aurora A. Immunofluorescence analysis also showed that excess centrosomes harbored an odd number of centrioles. Therefore, the authors hypothesized that BPA causes centrioles to over-duplicate by disrupting the level of centrosomal proteins. Indeed, the expression of *SASS6*, a core component of centrosome duplication, was significantly increased. BPA-mediated overexpression of *SSAS6* could provide excess templates for the formation of procentrioles, causing over-duplication of centrioles. In other studies, supernumerary centrosomes were detected in BPA-treated prostate cancer cells [14,217]. Here, the amplified centrosomes were intact because they contained two centrioles, as shown by co-immunostaining with the PCM-marker γ-tubulin and the centriole marker centrin, suggesting that the underlying mechanism of CA is centrosome duplication rather than centriole splitting [217]. Importantly, the authors showed that centrosome duplication occurred at an earlier time point in BPA-treated prostate cancer cells compared with control cells and that the number of centrioles increased significantly after treatment [14]. Therefore, it is possible that BPA dysregulates the timing of centrosome duplication. Consistent with this hypothesis, the expression of key G1-regulators such as CDK6 and cyclin D1 was increased, while the levels of the CDK-inhibitors p27 and p53 were decreased. In addition, NPM was found to be released from the single (unduplicated) centrosome earlier than in control-treated cells, which may allow another round of centriole duplication later during G1-phase or at the beginning of S-phase [14]. Interesting work in female ACI rats has shown that E2-induced mammary tumors are excessive in CA both in number and size [17,216]. Estrogen treatment significantly increased the expression and activity of centrosomal proteins such as Aurora A, centrin, cyclin E/CDK2, in these tumors [17,216], and overexpression of cyclin E is frequently observed in all types of human tumors [218]. The authors suggest that estrogen causes uncoupling of the centrosome and eukaryotic cell cycle, resulting in centriole re-duplication [216]. By contrast, by affecting *AURKA* and *CETN* expression, estrogens may impair centriole formation by altering the interaction of centriolar proteins, causing centrioles to over-duplicate [17,41,92].

Despite different underlying mechanisms, these results suggest that CA is an early event in pervasive and primary tumors that lead to chromosomal instability [17,216]. Consistent with this, estrogen-induced hamster kidney tumors showed significantly increased expression levels and kinase activity of Aurora A and B, linking estrogen to CA, chromosomal instability, aneuploidy, and tumor progression [20]. In addition, treatment of MCF7 cells with 2-MeOE2 caused many spindle abnormalities, including spindles with multiple poles. However, it is not clear whether these poles reflect functional centrosomes, as the cells were analyzed with α-tubulin rather than centrosome and/or centriole markers. Nevertheless, the authors postulated that 2-MeOE2 could selectively inhibit the interaction of ε-tubulin and other centrosomal proteins, thus impairing the proper recruitment of centrosomal components [219]. Disruption of ε-tubulin could interfere with proper centriole assembly or duplication of centrioles because interaction of ε-tubulin with α/β-tubulin of B- and C-tubules is required for centriole formation [44,45]. It follows that over-duplication of centrioles could be a possible mechanism of estrogen-induced CA.

### 6.2. Premature Centriole Disengagement

In addition to centriole over-duplication, the studies by Kim et al. also provided evidence for premature disengagement of centrioles as an important underlying mechanism of BPA-caused centrosome defects. The authors demonstrated an increased number of CNAP-1 foci when breast cancer cells were treated with BPA [13]. Since CNAP-1 is localized only at the free proximal ends of centrioles, BPA appears to cause premature centriole disengagement, allowing centrioles to reduplicate. In rat embryos, the environmental toxicant 2,3,7,8-tetrachlorodibenzo-p-dioxin (TCDD) modifies the number and localization of γ-tubulin foci at the compaction-stage of meiosis. Exposure to TCDD resulted in large γ-tubulin aggregates, indicating disruption of centrosome separation. However, no definite conclusions can be drawn because centriolar markers were not considered in this study [220].

### 6.3. Centrosome Amplification

Many research groups have observed an increase in spindle disorientation and organization upon treatment with estrogenic substances. For example, studies of V79 treated with a range of estrogenic substances have shown that the cells form multiple centrosomes and aberrant spindles [15,16]. Indeed, treatment with E2, BPA, and DES significantly disrupted the MT network and increased the frequency of cells with multipolar spindles. The cells predominantly had three centrosomes, leading to failure of spindle formation and multipolar cell divisions [15]. By contrast, treatment with estrone did not induce CA, leading to the conclusion that the 17-hydroxyl group may be required to induce supernumerary centrosomes [16]. Consistent with these results, treatment of V79 and human lymphoblastoid cell lines with low-dose BPA and rotenone resulted in supernumerary centrosomes, predominantly tripolar spindles, and CREST-positive micronuclei, demonstrating the presence of kinetochores and thus functional chromosomes rather than DNA damage [221]. Moreover, HPV16 E7 and E6 oncogenes increase centrosome copy number in estrogen-induced cervix carcinogenesis [222]. In mouse oocytes, treatment with 2-MeOE2 or BPA disrupted the distribution of pericentrin, resulting in severe spindle abnormalities and abnormal organization of spindle poles [223,224]. The meiotic progression delayed by BPA could be due to the displacement of pericentrin along the spindle microtubules, leading to disorganization of the spindle apparatus. Despite these defects, chromosomes were generally correctly arranged in the mid-plane [223]. Oocytes exposed to 2-MeOE2 showed similar patterns of mitotic spindles. The number of small and large MT asters and multipolar spindles was observed, indicating disruption of MT kinetics and organization. Pericentrin-positive centrosomes failed to organize properly because they were located in the periphery of the spindle body resulting in unaligned chromosomes and failure of congression [224]. However, in these studies, it remained unclear whether these aberrations were due to abnormalities in centrosome integrity because they used γ-tubulin or pericentrin, common PCM markers, for their analysis. Since mainly three centrosomes were observed in these studies, the underlying mechanisms are PCM fragmentation, centriole over-duplication, or premature centriole disengagement rather than cytokinesis failure.

### 6.4. Disruptive Microtubule Assembly and Dynamics

Estrogenic substances have been shown not only to affect centrosome integrity but also to interact directly with microtubules. Microtubules are key components of the cytoskeleton and mitotic spindle. Their polymerization dynamics are tightly regulated both spatially and temporally. There are three main tubulin binding sites: the paclitaxel site, the *Vinca* domain and the colchicine domain [225]. MT-active agents are either able to stabilize or destabilize MTs, thereby increasing or decreasing MT polymer mass, or suppress MT dynamics [226]. The ability of estrogens to disrupt MT assembly has been observed for nearly four decades. In 1986, Wheeler et al. first showed that estrogens can mediate aneuploidy by interacting with microtubules in Chinese hamster cells [227]. Since then, much research has been conducted. For example, in a study in a cell free-system, estrogens of different classes were examined for their ability to disrupt MT assembly. Estrogens such as Z, Zdienestrol, indanestrol, or threohexestrol, DES and BPA inhibited MT polymerization, whereas E2, genistein, daidzein and zearaleone did not. However, these might differ in intact cells [228]. Nevertheless, many studies have provided evidence that various estrogens induce disruption of MTs in vitro. This includes a study that examined the MT network in V79 cells in culture and Sertoli cells in whole animal systems after treatment with various environmental estrogens. In V79 cells, BPA, p-Nonylphenol, p-Octylphenol, p-Pentylphenol, E2, di-n-butyltin, dichloride, tri-n-butyltin chloride, and tetrabutyltin induced abnormal microtubule networks. Phthalate esters, on the other hand, showed no interfering activity in V79 cells. In the whole-animal system, none of these estrogenic chemicals exhibited disruptive effects [229]. Similarly, Aizu-Yokota et al. showed that several natural estrogens interfere with MTs in V79 cells. In the presence of the cytoskeletal drug taxol, which targets β-tubulin, E2-induced microtubule disruption was inhibited. Therefore, the authors interpreted that E2 is able to compete with taxol-promoted MT assembly. Preincubation of the transcription inhibitor actinomycin D and a translational inhibitor, cycloheximide, also demonstrated that the interference of estrogens with MTs does not require specific genomic stimulation. The structural basis of the various estrogens was shown to be essential for their ability to disrupt MTs. Their results suggest that 3-hydroxyl, 3-alkyl ether, and/or 3-methyl groups at C-3 contribute to MT-disruptive activity. They demonstrated that 2-MeOE2 exhibited the strongest MT-disruptive activity [230]. This is in agreement with the results of D’Amato et al. who showed that 2-MeOE2 interacts at the colchicine site, thereby inhibiting the polymerization of tubulin [231]. These MT-interrupting properties of 2-MeOE2 are thought to inhibit angiogenesis and suppress tumor growth [232,233,234]. By contrast, a study on MCF7 cells argues that the antimitotic and antiangiogenetic effects of 2-MeOE2 are probably not due to depolymerization of MTs, but rather to suppressed MT dynamics [219]. Like 2-MeOE2, DES is thought to bind to tubulin at the colchicine binding site [235]. A low dose of DES resulted in inhibition of depolymerisation of intact MTs, which promoted MT formation, whereas at high concentration polymerization was inhibited [236]. Furthermore, DES has been shown to inhibit MT assembly in a dose-dependent manner in a prostatic tumor cell line, resulting in metaphase-arrested cells [237]. Consistently, a detailed study of the interaction of DES with tubulin revealed a number of unique sets of properties. The results confirmed the competitive inhibitory properties of DES towards colchicine binding. Moreover, in the presence of microtubule-associated proteins (MAPs) Tau or MAP2, DES inhibited MT assembly [238]. In contrast to the protective effects of 2-MeOE2, the ability of DES to disrupt MTs is considered to underlie its carcinogenicity by promoting chromosomal instability and aneuploidy [239,240,241]. It is worth noting that DES was used for a long time to treat prostate cancer but was then abolished because of its toxicity [242]. The synthetic estrogens BPA, BP4 and BP5 also interact with microtubule proteins and interfere with MT formation in vitro. In a cell-free system, BPA appears to irreversibly alter the conformation of tubulin; however, in V79 cells, the effects on MTs were reversible after removal. Disruption of cytoplasmic MTs and mitotic spindles by these bisphenols resulted in CREST-positive micronuclei, which serve as a sign of w-CIN [243]. Disruptive effects of bisphenols on MTs were also observed in prostate cancer cells [14]. In an assay of MT-aster formation, the majority of cells treated with different bisphenols exhibited MT-asters, indicating changes in MT dynamics. Upregulation of the centrosomal protein CEP350, which is involved in MT anchoring and elongation, was also found [244]. Although the function of CEP350 was investigated in the context of MT stability, CEP350 has also been shown to be involved in the growth and stability of procentrioles [245]. Whether these MT-disrupting activities affect aneuploidy or occur in vivo remains the subject of future research, as this ability of estrogens does not necessarily correlate with their hormonal carcinogenesis. For example, 17α-estradiol, which is hormonally less active and non-carcinogenic, may interfere with MTs to the same extent as DES or E2 [18,246]. Estrone, which has been associated with proliferation and tumorigenesis, also has no effect on MTs [247]. Nevertheless, it is evident that estrogenic substances can affect MT dynamics and thus disrupt cell and mitotic spindle organization.

## 7. The Estrogen-Receptor-Centrosome Axis at a Glance

Accumulating evidence suggests that natural and environmental estrogens are capable of affecting the proper assembly of the mitotic spindle by interfering with centrosome integrity or microtubule assembly. However, whether these effects involve direct interference with centrosomal structures (e.g., centrioles) or with receptor signaling is largely unresolved and remains to be the subject of future research. As described in Section 5.1, estrogens mediate their effects by binding primarily to three estrogen receptors ERα, ERβ and GPER1, which regulate distinct functions. Given experimental studies showing that ERs and GPER1 physically localize to or interact with the centrosome and interact with centrosomal kinases, a potential estrogen receptor/centrosome-axis may exist [248,249,250,251,252]. Indeed, ERα has been shown to localize to the centrosome and the spindle during mitosis, thereby regulating chromosome alignment and spindle dynamics [248]. The localization of ERα to centrosomes appears to depend on Aurora A, Aurora B, and Plk1 [249]. In addition, ERβ has been reported to interact with the mitotic spindle assembly checkpoint protein, Mad2, highlighting its importance in cell cycle regulation [250]. In vascular smooth muscle cells, the activation of GPER1 has been associated with the induction of multipolar mitotic spindles [253]. Accordingly, when investigating a downstream pathway for estrogen-induced CA, it is critical to consider the estrogen receptor in question. Previous studies in female ACI rats suggested a role of ERα in estrogen-induced CA, as E2-induced mammary gland tumors were prevented by the concomitant administration of the selective estrogen receptor modulator tamoxifen [17]. Similarly, the effects of BPA and its analogues in prostate cancer cells are dependent on ERα [14]. By contrast, the interference of BPA with centrosome-associated proteins in breast cancer cells appears to be ERα-independent, as BPA did not affect the localization of ERα to the centrosome and mitotic spindle. In fact, maldistribution of chromosomes was induced in both ER-positive and ER-negative breast cancer cells [13]. Moreover, GPER1 has been reported to increase cAMP production and to activate protein kinase A (PKA), and low-dose BPA has been shown to induce PKA via GPER1 [252,254]. Members of the protein kinase (PK) family are known to regulate cell growth or cell cycle progression [255]. In fact, PKA is associated with several centrosomal components, as the anchoring of PKA to the centrosome by pericentrin and AKAP450/CG-NAP is an important step in centrosome function [256,257]. AKAP450/CG-NAP-anchored γTuRC is involved in microtubule nucleation and increases the number of centrosomes by recruiting cyclinE-cdk2 [258]. In addition, PKA and Aurora A have been shown to phosphorylate centrin during the G2/M phase of the cell cycle [41,92] and aberrant phosphorylation of centrin has been detected in breast cancer with centrosome amplification [156]. *AURKA* and *CETN* expression has been shown to be modulated by estrogen in ACI rats [17]. Although the authors suggested a role of ERα, it is possible that the GPER1-PKA-axis also has important functions, since GPER1 is also expressed in mammary glands. Indeed, PKA has been shown to induce changes in the conformation of ERα in breast cancer via GPER1 [259]. Moreover, endogenous protein kinase C (PKC) has been described to bind directly to pericentrin at the centrosome. Disruption of this interaction has been shown to lead to microtubule disorganization and excess centrosomes, likely due to cytokinesis failure [260]. Since MeOE2 and BPA have been shown to disrupt the distribution of pericentrin in mouse oocytes [223,224], it is possible that this is due to the interaction of PKC-pericentrin. Overall, estrogens and environmental estrogens could affect centrosomal integrity by altering the interaction of ERs with the centrosome or triggering centrosome-associated kinases via GPER1.

## 8. Conclusions and Future Perspective

It is evident that centrosomes are critical for genomic stability, as they contribute to correct chromosome segregation. Structural and numerical centrosome defects that promote erroneous microtubule-kinetochore attachments can manifest in w-CIN and aneuploidy, both of which are hallmarks of human cancer. Therefore, defects in centrosome integrity are likely to be a cause of tumor development. Increasing evidence suggests that estrogenic substances and their receptors have important functions in human carcinogenicity and tumor progression, with centriole over-duplication, premature centriole disengagement, and perturbed microtubule dynamics as possible tumor-promoting causes. Understanding how estrogen hormones affect centrosome homeostasis at the molecular level would therefore be a promising avenue of research, not least with regard to the effects of low doses and non-monotonic dose responses that follow unpredictable or atypical patterns (i.e., non-linear dose–effect relationships in which increasing doses do not result in increased effects across the entire concentration range) [261,262]. Indeed, concentration-response curves of cell cultures treated with BPA showed non-monotonic effect relationships with a maximum number of cells with supernumerary centrosomes at 10-100 pM decreasing at lower and also at higher concentrations [14,217]. This implies that effects of low (physiological) concentrations of hormones and EDCs may be significantly different from those of high (toxicological relevant) doses and that the effects of higher doses do not necessarily predict the effects of low doses. Concentrations of (xeno)estrogens in the micromolar to millimolar range have been shown to affect cell cycle progression and lead to inhibition of cell growth or even cytotoxicity [15,16,227,239], not least due to inhibition of microtubule polymerization [228,237,240,243]. Concentrations that rigorously inhibit cell growth would therefore have no effect on the establishment of centrosome defects and are unlikely to be important for tumorigenesis. By contrast, the concentrations that trigger mild CA and w-CIN are much more interesting in terms of carcinogenesis because they allow cell survival and therefore, pose a much higher risk of carcinogenesis.

To elucidate mechanism(s) behind CA triggered by low hormone concentrations, techniques such as (phospho)proteomics, proximity-dependent biotin identification (BioID [263]), and superresolution microscopy by using Photoactivated Localization Microscopy/Stochastic Optical Reconstruction Microscopy (PALM/STORM) could be powerful tools to fill the knowledge gap on the hormonal origin of centrosome defects.

## Data Availability

Not applicable.
